# Comparison of post-treatment recurrence between ranibizumab injection and laser photocoagulation for type 1 retinopathy of prematurity

**DOI:** 10.1186/s12886-023-02886-5

**Published:** 2023-04-04

**Authors:** Jing-Ke Cao, Tao Han, Hong-Yi Tang, Sheng Zhang, Zong-Hua Wang, Zhi-Chun Feng, Qiu-Ping Li

**Affiliations:** 1 The Second School of Clinical Medicine, Southern Medical University, Guangzhou, 510515 China; 2 Department of Neonatology, Senior Department of Pediatrics, The Seventh Medical Center of Chinese PLA General Hospital, NO.5 Nanmencang, Dongcheng District, Beijing, 100700 China; 3 The Affiliated Yantai Yuhuangding Hospital of Qingdao University, Yantai, 264000 China; 4 Department of Ophthalmology, the Seventh Medical Center of Chinese PLA General Hospital, Beijing, 100700 China

**Keywords:** Ranibizumab, Laser photocoagulation, Retinopathy of prematurity, Treatment

## Abstract

**Objective:**

To compare post-treatment recurrence between ranibizumab injection *and* l*aser* photocoagulation (LP) for type 1 retinopathy of prematurity (ROP), and explore the associated risk factors.

**Methods:**

The clinical data of ROP infants treated with LP or ranibizumab in a NICU of China from October 2007 to November 2021 were retrospectively analyzed and compared, such as general condition, degree of ROP, therapeutic effectiveness and post-treatment recurrence. The dependent variable was recurrence after ROP treatment. Univariate and regression analysis of risk factors was performed.

**Results:**

Of the 298 ROP infants (556 eyes), 58% of the eyes were treated with LP and the other 42% with ranibizumab. There was no significant difference in gestational age at birth, birth weight, sex, delivery mode, prenatal corticosteroids, ROP diagnosed before admission or after admission, and the duration of oxygen therapy between the two groups. However, the ratio of type 1 ROP and aggressive retinopathy of prematurity (A-ROP) in ranibizumab group was higher than that in LP group. The number of treatments, recurrence rate and recurrence interval in ranibizumab group were higher than those in LP group. However, there was no difference in the recurrence rate between the two groups after stratified analysis by the lesion area and the presence or absence of A-ROP. There was no significant difference in the final lesion regression between the two groups. Regression analysis showed that plus disease and ROP located in zone I were independent risk factors for post-treatment recurrence.

**Conclusion:**

There is no significant difference in the recurrence rate of ROP between ranibizumab injection and LP, and recurrence is mainly related to the severity of ROP. In half of our patients treated with A-ROP recurrences occur.

## Introduction

Retinopathy of prematurity (ROP) is a potentially blinding disease characterized by abnormal retinal neovascularization, affecting premature infants and very low birth weight infants, and remaining the main cause of blindness in children worldwide [1]. Laser photocoagulation (LP) is defined by the Early Treatment for Retinopathy of Prematurity (ET-ROP) study [2] as the gold standard for the treatment of type 1 ROP in that it can destroy the peripheral retina. However, laser therapy is also associated with some demerits such as visual field reduction, myopia and exudative detachment [3]. In recent years, anti-vascular endothelial growth factor (VEGF) agents have been widely used as an alternative to treat severe ROP, knowing that they can prevent or reduce pathological neovascularization and maintain retinal integrity. Previous studies have shown that anti-VEGF agents have a better therapeutic effect on ROP compared with conventional laser therapy, especially for zone I stage 3 ROP [4, 5]. Bevacizumab is the first anti-VEGF drug for ROP, but these drugs may cause temporary suppression of growth factors such as systemic VEGF [6–8]. Some studies suggest that bevacizumab treatment may be associated with adverse neurodevelopmental outcomes [9]. Compared with bevacizumab, ranibizumab is considered a safer treatment modality because of a smaller molecular weight with a shorter half-life and faster clearance in the body, thus reducing the risk of neurological developmental defects [10, 11]. Therefore, it has been more widely used in recent years.

However, post-treatment recurrence is a common complication both for LP and anti-VEGF therapies. Some studies have shown that the recurrence rate in infants treated by ranibizumab injection is higher than that by LP [12, 13], but other studies reported controversial conclusions [13]. The risk factors of recurrence in LP or ranibizumab therapy are also unclear. The purpose of this study was to explore the therapeutic efficacy, especially the recurrence rate of ranibizumab intravitreal injection and LP in the treatment of ROP by retrospectively analyzing the clinical data of 298 ROP children of Han ethnicity, and explore associated risk factors in Chinese children.

## Materials and methods

### Subjects of the study

This study protocol follows the principle stated in the Declaration of Helsinki, and the study was approved by the research ethics board of the Seventh Medical Center of Chinese PLA General Hospital (Beijing, China), with a waiver of informed consent from this ethics board because of the retrospective design of the study. The subjects were ROP infants treated with LP or ranibizumab in the NICU of the Seventh Medical Center of Chinese PLA General Hospital (Beijing, China) consecutively from October 2007 to November 2021.

### Inclusion and exclusion criteria

The inclusion criteria were premature infants diagnosed with type 1 ROP by binocular indirect ophthalmoscopy or RetCam II system and initially treated with ranibizumab or LP. The exclusion criteria were premature infants who were initially treated with other methods such as cryotherapy and vitrectomy, and ROP infants who had progressed to stage 4 and above before the first treatment. Three included infants were lost to follow-up after treatment.

### Fundus examination

**Fig. 1:**
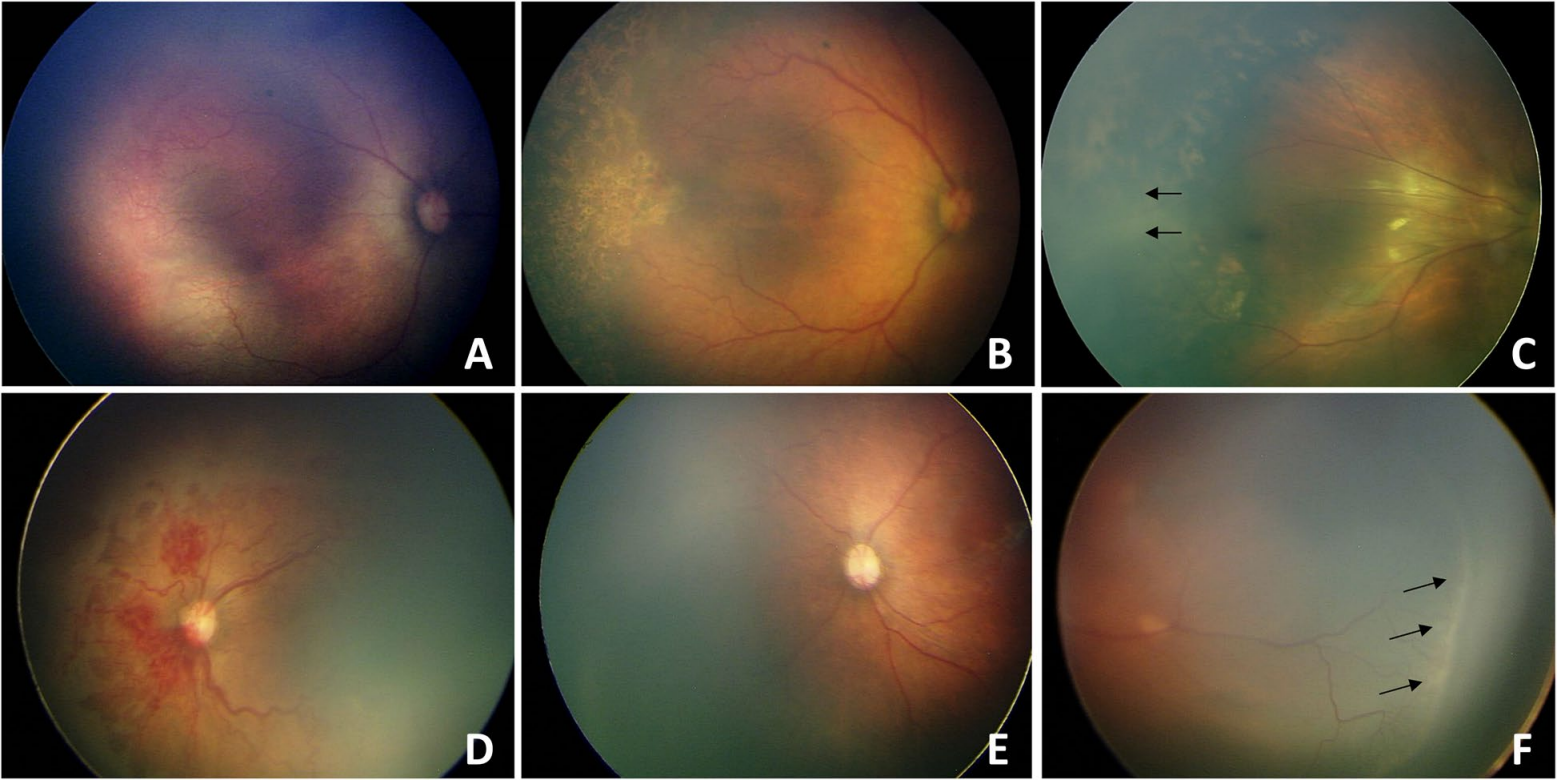
Fundus examination of retinopathy of premature. **A**-**C** Fundus pictures of an infant with zone 2 plus ( +) stage 1 ROP before, after laser treatment, and at the time of recurrence. **A**. Before laser treatment; **B**. Lesion regressed of 4w after laser treatment; **C**. Fiber proliferation in peripheral laser area 9w after treatment (arrow), and traction of retina; **D**-**F** Fundus pictures of an infant with A-ROP before, after ranibizumab injection treatment and at the time of recurrence; **D**. Before ranibizumab injection treatment; **E**. Lesions regressed significantly 3w after treatment; **F**. Reexamination at 7 weeks after treatment showed peripheral ridge lesions (arrows)

According to the Chinese National Guidelines on ROP screening [14], the first fundus examination was performed on premature infants with GA < 34W and / or BW < 2 kg 4–6 weeks after birth. Prior to the examination, the infants were deprived of food and water for 2 h. The pupils were dilated with Mydriatic Eyedrops (0.5% tropicamide, 3–4 times for 10 min per time), followed by application of local anesthetic eye drops (0.4% oxybuprocaine). Ophthalmologic examination was performed at the NICU using the + 28D binocular indirect ophthalmoscope or RetCam II digital camera (Clarity Medical systems, Inc., USA). Ofloxacin (0.3%) was applied for contact between the camera lens and the cornea when RetCam II digital camera was used. Recurrence, regression of ROP was defined according to the criteria set by the International Classification of Retinopathy of Prematurity (ICROP3) [15]. According to the results of the previous examination, fundus reexamination was arranged every 1–2 weeks (Fig. 1).

### Operative method

ROP was treated according to the criteria set by the Early Treatment for Retinopathy of Prematurity Cooperative Group (ET-ROP) [2]. LP or intravitreal injection of ranibizumab was performed within 72 h after diagnosis of threshold ROP or type 1 pre-threshold ROP. Threshold ROP was defined as type 3 ROP with lesions over five continuous clock hours or eight accumulated clock hours. Type 1 pre-threshold ROP was defined as zone I, any stage ROP with plus disease, stage 3 ROP without plus disease, or zone II, stage 2 or 3 ROP with plus disease. Stage 4 or 5 ROP was treated with vitrectomy. Both LP and ranibizumab injection were completed by the same ophthalmologist. The LP procedure was performed as follows: the family members signed informed consent on surgery and anesthesia. All LP procedures were performed under topical anesthesia combined with general anesthesia with an anesthetist on standby. Laser used was diode 810 nm (Oculight SLx, Iridex Co, LA, and USA). Power settings were titrated to achieve a gray-white burn of moderate intensity, and spots were applied in a confluent manner to cover the entire avascular retina from the ridge to the ora serrata. While the ranibizumab injection procedure was performed as follows: the eye was dilated in advance and topical drop anesthesia was applied with 0.4% Obucaine Hydrochloride Eye Drops before operation. The infant was laid flat with the head fixed. Then, the eyes were disinfected locally, the eyelid was opened with the eyelid opener, the corneoscleral edge of the eye was clamped with the forceps, the corneoscleral edge of the other side was fully exposed, and the needle was inserted vertically 1 mm from the corneoscleral edge, followed by slow injection of 0.025 ml (0.25 mg) ranibizumab (Novartis, Switzerland). After slow withdrawal of the injection needle, a cotton swab was applied with pressure onto the injection site to ensure no blood and drug flowing out. The ophthalmic conjunctival sac was coated with diapaxin ointment and covered with sterile gauze. After injection, levofloxacin eye drops were applied to prevent eye infection.

### Follow-up observation

The treated eyes were followed up closely weekly or more frequently when necessary for signs of regression or need for further intervention, until lesions regressing.

### Data collection

Depending on the method of initial treatment, the children were divided into a ranibizumab group and a LP group. Data in both groups were collected, including birth weight (BW), gestational age (GA), sex, mode of delivery, prenatal dexamethasone, whether transfer for ROP, duration of oxygen supplement, time of initial treatment, classification of ROP, whether regress after initial treatment, number of treatment and cure rate.

### Statistical analysis

Data were analyzed by SPSS 18.0. Counting data including GA at birth, BW and the duration of oxygen therapy are expressed as the mean ± standard deviation (`x ± s). The mean comparison between the two groups was conducted by *t* test, and the rate comparison between the two groups was conducted by Chi-square test. The relevant factors associated with lesion regression were analyzed by *t* test and Chi-square test first, and finally by logistic regression analysis, with P < 0.05 as the difference having statistical significance.

## Results

### Basic information

A total of 298 ROP infants were included in this study, of whom 124 (241 eyes) received ranibizumab injection, accounting for 42%, and the other 174 (315 eyes) received LP. There was no significant difference between the two groups in terms of GA at birth, BW, sex, mode of delivery, prenatal corticosteroids, ROP diagnosed before admission or after admission, and the duration of oxygen therapy (Table 1).

**Table 1:** Comparison of the basic clinical data between ranibizumab injection and LP groups

Items	Ranibizumab (*n* = 124)	LP (*n* = 174)	t/χ2 values	*P* values
Gestational age (w)	29.03 ± 1.74	29.16 ± 2.03	0.559	0.577
Birth weight (g)	1191.81 ± 265.40	1209.75 ± 293.78	0.541	0.589
Sex(male)	73(58.87%)	107(61.49%)	0.208	0.648
C-section	60(48.39%)	67(38.51%)	2.891	0.089
*Prenatal corticosteroids*	30(24.19%)	29(16.67%)	3.207	0.073
*ROP diagnosed before admission*	96(77.42%)	122(70.11%)	1.967	0.161
*Duration of oxygen therapy(d)*	37.10 ± 28.12	31.94 ± 24.33	1.573	0.117

*LP* laser photocoagulation, *ROP* retinopathy of prematurity

### Differences in ROP categories and outcomes between Ranibizumab and LP groups

According to the lesion division, the proportion of zone I ROP was 58.06% in ranibizumab group *vs.* 17.24% in LP group (c2 = 33.341, P < 0.05). Compared with LP group, the proportion of aggressive retinopathy of prematurity (A-ROP) in ranibizumab group was higher (29.9% *vs.*13.0%, P < 0.05); the postmenstrual age (PMA) of initial treatment was lower; the post-treatment recurrence rate and the number of treatments required were higher; the recurrence interval was longer. But there was no significant difference in the final lesion regression rate between the two groups (Table 2).

**Table 2:** Differences in ROP categories and outcomes between the Ranibizumab and laser groups

Items	Ranibizumab (eyes) (*n* = 241)	LP (eyes) (*n* = 315)	t/χ2 values	*P* values
ZoneIROP	141(58.5%)	58(18.4%)	95.506	< 0.001
A-ROP	72(29.9%)	41(13.0%)	23.967	< 0.001
PMA of initial treatment (w)	35.53 ± 2.39	37.43 ± 3.28	7.580	< 0.001
Duration of follow-up (m)	19.6 ± 4.8	20.1 ± 5.3	1.148	0.0014
Recurrence rate	44(18.3%)	30(9.5%)	9.026	0.0026
Number of treatments	1.216 ± 0.48	1.10 ± 0.32	3.098	0.002
Recurrence interval (w)	9.3 ± 5.1	5.6 ± 3.7	3.406	0.001
Final lesion regression rate	236(97.92%)	302(95.87%)	1.836	0.175

*LP* laser photocoagulation, *ROP* retinopathy of prematurity, *A-ROP* aggressive retinopathy of prematurity

### Comparison of ROP recurrence rates in terms of severity

There were 199 eyes with zone I ROP, of which 38 eyes recurred, with a recurrence rate of 19.10%. There were 357 eyes with zone II, of which 36 eyes recurred, with a recurrence rate of 10.08%. The recurrence rate of ROP in zone I was significantly higher than that in zone II (c2 = 8.993, P = 0.0027). However, when the recurrence rates of the two treatments were statistically analyzed according to different lesion zones, it was found that there was no statistically significant difference between the two groups (Table 3). Of all 556 eyes treated, 113 eyes had A-ROP lesions and 56 eyes recurred, with a recurrence rate of 49.56%. There were 443 eyes with non-AROP lesions and only 18 eyes recurred, with a recurrence rate of 4.06%. The recurrence rate of A-ROP lesions was higher than that of non-AROP lesions (c2 = 161.507, P < 0.001). However, there was no significant difference in the recurrence rate of A-ROP and non-AROP lesions treated by the two treatment methods (P > 0.05) (Table 4).

**Table 3:** Comparison of the recurrence rate in zone I ROP and zone II ROP treated by ranibizumab or LP

Treatment	zone I ROP		zone II ROP	
	Regression	Recurrence	Regression	Recurrence
Ranibizumab group, eyes(%)	111(78.72%)	30(21.28%)	86(86.0%)	14(14.0%)
LP group, eyes(%)	50(86.21%)	8(13.79%)	235(91.4%)	22(8.6%)
c^2^	1.4897		2.349	
P	0.222		0.125	

*LP* laser photocoagulation, *ROP* retinopathy of prematurity

**Table 4:** Comparison of the recurrence rate in A-ROP and non-AROP treated by ranibizumab or LP

Treatment	A-ROP		Non-AROP	
	Regression	Recurrence	Regression	Recurrence
Ranibizumab group, eyes (%)	36(50.0%)	36(50.0%)	161(95.27%)	8(4.73%)
LP group, eyes(%)	21(51.22%)	20(48.78%)	264(96.35%)	10(3.65%)
c^2^	0.0155		0.3151	
P	0.901		0.575	

*LP* laser photocoagulation, *ROP* retinopathy of prematurity, *A-ROP* aggressive retinopathy of prematurity

### Analysis of independent risk factors associated with ROP recurrence

Logistic regression analysis on risk factors associated with ROP recurrence after initial treatment showed that plus disease and zone I lesion were the main risk factors associated with recurrence after initial treatment, and GA at birth, BW and first treatment choice were not independent risk factors associated with recurrence (Table 5).

**Table 5:** Regression analysis on risk factors of recurrence after initial treatment

Items	B	S.E,	Wals	df	Sig	Exp (B)
Gestation age (w)	-.015	.088	.027	1	.869	.986
Birth weight	-.001	.001	1.159	1	.282	.999
Plus disease	1.124	.454	6.140	1	.013	3.078
Zone I ROP	.587	.277	4.496	1	.034	1.799
First treatment choice	-.313	.280	1.248	1	.264	.731

*ROP* retinopathy of prematurity

## Discussion

In this study, we observed the short-term outcomes of LP and ranibizumab injection for the treatment of type 1 ROP through retrospective analysis of a large single-center sample. It was found that ranibizumab injection and LP were equally effective for type 1 ROP. There was no significant difference in the final lesion regression rate between the two groups, but the post-treatment recurrence rate in ranibizumab group was higher than that in LP group. However, there was no difference in the recurrence rate between the two groups after stratified analysis by the lesion area and the presence or absence of A-ROP. We also found that plus disease and ROP located in zone I were independent risk factors for post-treatment recurrence by regression analysis.

The Department of Neonatology of the Seventh Medical Center of Chinese PLA General Hospital is the largest neonatal treatment center in China and the ROP treatment center in North China. Of the 298 ROP infants included in this study, 218 were transferred from hospitals of various provinces in North China. There was no significant difference in basic information of the included infants in terms of GA at birth, BW, sex and the delivery mode between LP and ranibizumab groups. It was found in our study that the proportion of zone I ROP and A-ROP in ranibizumab group was higher than that in LP group, indicating that the lesions in ranibizumab group were more serious than those in LP group. As it was a long-term retrospective study, and zone I ROP and A-ROP were more likely to be treated with ranibizumab in the later stage of the study, there was a huge difference in disease severity between the two groups. The initial treatment time between the two groups was also significantly different. The average PMA of initial treatment in ranibizumab group was 35.53 weeks *vs.* 37.43 weeks in LP group. Two studies in Turkey also found that the first treatment time of ranibizumab and bevacizumab group was earlier than that of LP group [12, 16]. We believe that this difference in our study may be related to the higher proportion of zone I ROP and A-ROP in ranibizumab group, which reached the treatment threshold earlier. From the perspective of ROP pathogenesis, the initial retinal vasculature develops through vasculogenesis in the posterior pole from precursor cells that migrate out of the deep retina and into inner layers, and these precursor cells become angioblasts at approximately 15–22 weeks of gestation and form an inner vascular plexus that extends to approximately zone I [17]. By 34 weeks of PMA, the retinal vasculature completely extents to zone II [18], indicating that ROP occurs earlier in zone I than in zone II.

Laser therapy and anti-VEGF antibody injection are the mainstay of treatment for ROP at present, but both methods are associated with the risk of recurrence. In addition, controversy remains on which of the two methods has a lower recurrence rate. One early study evaluated the recurrence of stage 3 + ROP of infants at PMA of 54 weeks after being treated by bevacizumab or laser therapy, and found that the rate recurrence of zone I and posterior zone II combined in intravitreal bevacizumab group was significantly lower than that in conventional laser therapy group (6% *vs.* 26%), and the same result was found with zone I disease alone (6% *vs.* 42%), but there was no significant difference with zone II posterior disease alone between the two groups (5% *vs.* 12%) [4]. A recent randomized, open-label, superiority RAINBOW trial [19] assessed the efficacy and safety of intravitreal ranibizumab and LP for ROP for 24 weeks, and the result showed that the treatment success rate was 80% in 0.2 mg ranibizumab group, 75% in 0.1 mg ranibizumab group, and 66% in LP group. A retrospective study [20] showed that the recurrence rate after LP, intravitreal bevacizumab monotherapy, or intravitreal ranibizumab monotherapy for ROP was 18.0%, 10.0% and 20.8% respectively, showing no significant difference between them. However, many studies conducted with ranibizumab reported the different outcomes. Zhang et al. reported that the recurrence rate in ranibizumab group was significantly higher than that in LP group for zone II ROP [13]. In a comparative study on the effect of ranibizumab, bevacizumab and LP on ROP [12], Gunay et al. found that the recurrence rate of ranibizumab group was higher than that of bevacizumab and LP groups, which may be related to the different pharmacokinetic characteristics of bevacizumab and ranibizumab. Bevacizumab has a long half-life in the body and is cleared from the body at a slower rate than ranibizumab [6, 21, 22]. In our study, the recurrence rate in the ranibizumab group was significantly higher than that in LP group, and the number of treatments in the ranibizumab group was also higher than that in LP group. However, the severity of the lesion in each group was obviously different between Gunay’s and our study in that the proportion of zone I ROP in ranibizumab group of their series was significantly higher than that in LP group (63.6% *vs.* 12.3%), *vs.* 58.5% and 18.4% in our study. To clarify the impact of disease severity on the post-treatment recurrence rate, we divided the lesions into zone I and zone II, as well as A-ROP and non-AROP, and found that the recurrence rate in zone I and A-ROP was significantly higher than that in zone II and non-AROP (19.1% *vs.* 10.08%; 49.56% *vs.* 4.06%), and the difference between the two groups was statistically significant. The results indicated that the reason for higher recurrence rate in ranibizumab group was not only related to its short half-life, but also to severity of the disease. We further compared the effect of the two treatments on the recurrence rate in patients with the same degree of lesions, and found that the recurrence rate between the two groups was not statistically significant, showing no obvious disadvantage of ranibizumab therapy compared with LP in terms of the recurrence rate in patients with the same degree of lesions.

In addition, as the half-life of ranibizumab is shorter than that of bevacizumab, the recurrence interval should be theoretically shorter than that of bevacizumab. But the recurrence interval reported in different studies varied. The BEAT-ROP study showed that the mean recurrence interval after bevacizumab treatment was 16 weeks [4]. Hu et al. reported that it was 14.4 weeks [23]. Other studies reported that the recurrence interval of ranibizumab treatment was significantly shorter than that of bevacizumab. Feng et al. reported that the recurrence interval was 8.57 ± 3.73 weeks in their 331 cases (629 eyes) treated with ranibizumab [24]. Wong et al. reported that the recurrence interval was only 5–7 weeks in their 5 cases treated with ranibizumab [25]. Lyu et al. reported that the recurrence risk period of ranibizumab treatment was 2.5–12 weeks after initial treatment, with a peak risk at 8 weeks [26]. These different results may also be related to the time gap in follow-up across trials. A follow-up to at least 70 weeks PMA until complete involution of ROP with vascularization of zone III was recommended for patients treated with intravitreal bevacizumab monotherapy [27]. Lyu et al. suggested that at least 24-week follow-up period after receiving ranibizumab monotherapy or a 60-weeks follow-up period for PMA was preferred to short-term follow-up periods [26]. In our study, the mean interval from the initial treatment to recurrence was 9.3 weeks in ranibizumab group, which is similar to the finding of Feng’s [24]. It was found in our study that the recurrence interval of ranibizumab treatment was significantly higher than that of LP group (mean 5.6 weeks), suggesting that a longer follow-up period is required after ranibizumab treatment.

Our further multivariate logistic regression analysis showed that zone I ROP and plus disease were independent risk factors for recurrence. The different recurrence rates between ranibizumab and LP groups may be attributed to differences in these parameters rather than to differences in treatment methods in this study, and we also found that lesions in zone I and plus lesions were predictive factors of recurrence, which may be able to help develop treatment and follow-up strategies in advance. Therefore, strict and standardized follow-up observation should be carried out after treatment for zone I ROP and ROP with plus disease no matter what treatment method is adopted.

This study has some limitations. Firstly, selection bias may be unavoidable due to the retrospective nature of the study. In addition, as this research project lasted for a relatively long period, laser therapy was applied more frequently in the earlier years, and ranibizumab therapy was applied more frequently in recent years. Finally, this study only compared the short-term outcomes of these different therapies and did not evaluate their long-term impact on visual, brain and lung development.

In conclusion, our study provides more evidence-based information on the efficacy and short-term safety of ranibizumab in the treatment of ROP. The higher recurrence rate in ranibizumab group may be related to severity of the disease, but there was no significant difference in the recurrence rate between ranibizumab group and LP group in patients with the same degree of severity of the lesions. Zone I ROP and plus disease are independent risk factors for recurrence. Ranibizumab therapy is expected to become a first-line therapy for ROP, though its long-term impact on brain and lung development remains to be evaluated.

## Data Availability

Not applicable.

## References

[1] Taner A, Tekle S, Hothorn T, Adams M, Bassler D, Gerth-Kahlert C (2020). Higher incidence of retinopathy of prematurity in extremely preterm infants associated with improved survival rates. Acta Paediatr.

[2] Early Treatment For Retinopathy Of Prematurity Cooperative Group (2003). Revised indications for the treatment of retinopathy of prematurity: results of the early treatment for retinopathy of prematurity randomized trial. Arch Ophthalmol.

[3] Zou H, Fletcher-Morehouse L (2022). Exudative Retinal Detachment After ROP Laser Photocoagulation. Cureus..

[4] Mintz-Hittner H, Kennedy K, Chuang A, JTNEjom (2011). Efficacy of intravitreal bevacizumab for stage 3+retinopathy of prematurity. N Eng J Med.

[5] Mintz-Hittner H, Kuffel RJR (2008). Intravitreal injection of bevacizumab (avastin) for treatment of stage 3 retinopathy of prematurity in zone I or posterior zone II. Retina.

[6] Wei-Chi Wu, Shih Chia-Pang, Lien Reyin, Wang Nan-Kai, Chen Yen-Po, Chao An-Ning, Chen Kuan-Jen, Chen Tun-Lu, Hwang Yih-Shiou, Lai Chi-Chun (2017). Serum Vascular Endothelial Growth Factor After Bevacizumab Or Ranibizumab Treatment For Retinopathy Of Prematurity. Retina..

[7] Travassos A, Teixeira S, Ferreira P, Regadas I, Travassos A, Esperancinha F (2007). Intravitreal bevacizumab in aggressive posterior retinopathy of prematurity. Ophthalmic Surg Lasers Imaging.

[8] Kusaka S, Shima C, Wada K, Arahori H, Shimojyo H, Sato T, Fujikado TJTBjoo (2008). Efficacy of intravitreal injection of bevacizumab for severe retinopathy of prematurity: a pilot study. Br J Ophthalmol..

[9] Natarajan G, Shankaran S, Nolen T, Sridhar A, Kennedy K, Hintz S (2019). Neurodevelopmental Outcomes of Preterm Infants With Retinopathy of Prematurity by Treatment. Pediatrics..

[10] Castellanos MA, Schwartz S, Garcia-Aguirre G, Quiroz-Mercado H (2013). Short-term outcome after intravitreal ranibizumab injections for the treatment of retinopathy of prematurity. Br J Ophthalmol.

[11] Baumal C, Goldberg R, Fein J (2015). Primary intravitreal ranibizumab for high-risk retinopathy of prematurity. Ophthalmic Surg Lasers Imaging Retina.

[12] Gunay M, Sukgen EA, Celik G, Kocluk Y (2017). Comparison of Bevacizumab, Ranibizumab, and Laser Photocoagulation in the Treatment of Retinopathy of Prematurity in Turkey. Curr Eye Res.

[13] Zhang G, Yang M, Zeng J, Vakros G, Su K, Chen M, Li H (2017). Comparison Of Intravitreal Injection Of Ranibizumab Versus Laser Therapy For Zone II Treatment-Requiring Retinopathy Of Prematurity. Retina.

[14] Fundus Disease Group, Ophthalmology Branch of Chinese Medical Association. Guidelines for Retinopathy of Prematurity Screening in China, Article in Chinese. Chinese J Ophthalmol. 2014;50(12):933–35. 10.3760/cma.j.issn.0412-4081.

[15] Chiang MF, Quinn GE, Fielder AR, Ostmo SR, Paul Chan RV, Berrocal A (2021). International Classification of Retinopathy of Prematurity. Third Edition Ophthalmology.

[16] Kabatas EU, Kurtul BE, Altiaylik Ozer P, Kabatas N (2017). Comparison of Intravitreal Bevacizumab, Intravitreal Ranibizumab and Laser Photocoagulation for Treatment of Type 1 Retinopathy of Prematurity in Turkish Preterm Children. Curr Eye Res.

[17] Hartnett ME (2015). Pathophysiology and mechanisms of severe retinopathy of prematurity. Ophthalmology.

[18] Jang JH, Kim YC (2020). Retinal vascular development in an immature retina at 33–34 weeks postmenstrual age predicts retinopathy of prematurity. Sci Rep.

[19] Stahl A, Lepore D, Fielder A, Fleck B, Reynolds JD, Chiang MF (2019). Ranibizumab versus laser therapy for the treatment of very low birthweight infants with retinopathy of prematurity (RAINBOW): an open-label randomised controlled trial. Lancet.

[20] Ling KP, Liao PJ, Wang NK, Chao AN, Chen KJ, Chen TL (2020). Rates and Risk Factors for Recurrence of Retinopathy of Prematurity after Laser or Intravitreal Anti-Vascular Endothelial Growth Factor Monotherapy. Retina.

[21] Wu WC, Lien R, Liao PJ, Wang NK, Chen YP, Chao AN (2015). Serum levels of vascular endothelial growth factor and related factors after intravitreous bevacizumab injection for retinopathy of prematurity. JAMA Ophthalmol.

[22] Zhou Y, Jiang Y, Bai Y, Wen J, Chen L (2016). Vascular endothelial growth factor plasma levels before and after treatment of retinopathy of prematurity with ranibizumab. Graefes Arch Clin Exp Ophthalmol.

[23] Hu J, Blair MP, Shapiro MJ, Lichtenstein SJ, Galasso JM, Kapur R (2012). Reactivation of retinopathy of prematurity after bevacizumab injection. Arch Ophthalmol.

[24] Feng J, Qian J, Jiang Y, Zhao M, Liang J, Yin H (2017). Efficacy of Primary Intravitreal Ranibizumab for Retinopathy of Prematurity in China. Ophthalmology.

[25] Wong R, Hubschman S, Tsui IJR (2015). Reactivation of retinopathy of prematurity after ranibizumab treatment. Retina.

[26] Lyu J, Zhang Q, Chen CL, Xu Y, Ji XD, Li JK (2017). Recurrence of Retinopathy of Prematurity After Intravitreal Ranibizumab Monotherapy: Timing and Risk Factors. Invest Ophthalmol Vis Sci.

[27] Mintz-Hittner HA, Geloneck MM, Chuang AZ (2016). Clinical Management of Recurrent Retinopathy of Prematurity after Intravitreal Bevacizumab Monotherapy. Ophthalmology.

